# Frontiers of Ovarian Carcinosarcoma

**DOI:** 10.1007/s11864-023-01138-4

**Published:** 2023-11-08

**Authors:** Ayden Ismail, Sunyoung Choi, Stergios Boussios

**Affiliations:** 1 GKT School of Medicine, King’s College London, London, SE1 9RT UK; 2 Department of Medical Oncology, Medway NHS Foundation Trust, Windmill Road, Kent, Gillingham, ME7 5NY UK; 3 Faculty of Life Sciences & Medicine, School of Cancer & Pharmaceutical Sciences, King’s College London, London, SE1 9RT UK; 4 Kent Medway Medical School, University of Kent, Kent, Canterbury, CT2 7LX UK; 5 AELIA Organization, 9Th Km Thessaloniki-Thermi, 57001 Thessaloniki, Greece

**Keywords:** Ovarian carcinosarcoma, Malignant mixed Müllerian tumour, Chemotherapy, Cytoreductive surgery, Targeted therapy, Treatment

## Abstract

Ovarian carcinosarcoma (OCS), also known as a malignant mixed Müllerian tumour (MMMT), is a rare and aggressive form of cancer that accounts for less than 5% of ovarian cancers. It is characterized by high morbidity and mortality rates, with a median overall survival (OS) of less than 2 years. Several factors, including advancing age, nulliparity, reduced lactation rates, decreased use of oral contraceptive pills, genetic mutations in *BRCA* (breast cancer) genes, and the use of assisted reproductive technology, may increase the risk of OCS. Poor prognostic factors include an advanced stage at diagnosis, older age, lymph node metastasis, suboptimal surgical cytoreduction, the presence of heterologous features on histopathology, and increased expression of vascular endothelial growth factor (VEGF), tumour protein p53, and p53 alongside Wilms tumour 1 (WT1). The main treatment approach for OCS is cytoreductive surgery followed by platinum-based chemotherapy, although immunotherapy is showing promise. Homologous recombination deficiency (HRD) testing may enhance outcomes by enabling personalized immunotherapy and targeted therapies for specific patient groups, thereby reducing unnecessary side effects and healthcare costs. However, there is currently a lack of standardised treatment regimens for OCS patients, with most studies consisting of case reports and a shortage of suitable comparator groups. This article aims to provide clinicians with information on the epidemiology, risk factors, prognostic factors, and latest therapeutic advancements in OCS.

## Introduction

Ovarian cancer ranks as the fifth primary cause of cancer-related deaths in women. According to the GLOBOCAN study, there is a projected global surge of 55% in ovarian cancer cases and a 67% rise in mortality between 2012 and 2035 [1]. The major risk factors for ovarian cancer include a family history of the disease and genetic syndromes associated with it. Although factors like obesity, smoking, and a sedentary lifestyle are linked to an increased risk of ovarian cancer, they have not been established as definitive predisposing factors [2]. Endometriosis is directly correlated with certain subtypes of ovarian cancer, specifically clear cell and endometrioid ovarian carcinoma [3]. Currently, there are no effective screening tools available, and the cost-effectiveness analyses of screening programs have yielded mixed results [4]. More than two-thirds of women are diagnosed with advanced stages of ovarian cancer, leading to estimated 5-year survival rates ranging from 20 to 40%, whilst individuals diagnosed at an early stage experience a 5-year survival rate exceeding 90% [5, 6]. The conventional treatment approach involves cytoreductive surgery combined with platinum-based chemotherapy administered before or after the operation [7]. However, as many as 80% of patients experience a relapse within 12 to 18 months after completing the treatment, requiring first-line chemotherapy based on their platinum sensitivity. Specific subsets of patients may benefit from molecular targeted therapy, which provides improved treatment responses and reduced systemic toxicity. The inherent genomic and epigenomic diversity of ovarian cancer is mirrored at the protein level, presenting potential opportunities for discovering new pharmacological targets [8].

Histologically, ovarian cancer is categorized into epithelial and non-epithelial types. Within epithelial ovarian cancers (EOC), the World Health Organisation defines seven histological subtypes—serous, endometrioid, clear cell, mesonephric-like carcinoma, dedifferentiated carcinoma, mixed carcinoma, and carcinosarcoma [9]. Each subtype represents a unique entity with variations in clinical manifestations, genetic mutations, and treatment responses.

Ovarian carcinosarcoma (OCS), otherwise known as a malignant mixed Müllerian tumour, is a rare, aggressive type of epithelial ovarian neoplasm, accounting for less than 5% of ovarian malignancies [10, 11]. Histologically, OCS consists of both high-grade carcinomatous and sarcomatous elements [10, 12]. Although there have been reports indicating similarities between OCS and more common subtypes of EOC, OCS is considered to possess distinct clinical characteristics that set it apart and result in distinctive behaviour [10, 13•, 14]. It is an uncommon form of gynaecological cancer associated with high morbidity and mortality, and the prognosis continues to be dismal [14, 15]. According to several studies, OCS is reported to have a worse prognosis compared to other forms of ovarian malignancies, such as papillary serous ovarian carcinoma, in all International Federation of Gynaecology and Obstetrics (FIGO) stages [16–18]. Additionally, some patients may experience non-specific symptoms of ovarian cancer (gastrointestinal disturbance, bloating, and early satiety), whilst others may be asymptomatic, further contributing to poor survival via late clinical presentation and consequently a more advanced FIGO stage at diagnosis [19]. Whilst more common types of EOC are managed with cytoreductive surgery and neo- and/or adjuvant platinum-based chemotherapy, OCS has no established or standardised treatments, and large-scale prospective studies remain scarce [20, 21].

This article aims to provide clinicians with information regarding the epidemiology of OCS, risk factors associated with the disease, prognostic factors, and the evaluation of the latest developments in therapeutics.

### Epidemiology and risk factors

Globally, ovarian cancer is the fifth most common gynaecological cancer, accounting for approximately a quarter of a million diagnoses and more than 150,000 deaths each year [2, 22]. Higher incidence rates and mortality risks have been identified in more developed regions [2]. EOC is the most predominant type of ovarian cancer, whereas OCS represents a rare yet biologically unique ovarian cancer with a poor prognosis [23, 24]. According to a consensus review conducted by the Gynecologic Cancer InterGroup, the median overall survival (OS) for OCS is reported to be less than 2 years, and approximately 90% of OCS cases exhibit malignant spread [24].

In the UK, on average, more than a quarter of new ovarian cancer cases (28%) are diagnosed in patients aged 75 and over [25]. Similarly to most cancer types, incidence increases with age, as this largely reflects cell DNA damage accumulating over time. The average age of OCS onset is between 60 and 70 years of age [26]. Ovarian cancer incidence rates are projected to rise by 5% in the UK over the next two decades, which could be due to population growth and an ageing population, but also other risk factors such as increasing nulliparity rates and decreased lactation, decreasing use of oral contraceptive pills, and increasing use of assisted reproductive technology and mutations in *BRCA* genes [25, 27].

Detailed regional statistics for OCS incidence or distribution are unavailable due to their rarity, thus providing difficulty for critical analysis of regional differences [10, 11]. Racial disparities among 2866 patients with OCS were investigated by the Uniformed Services University, USA, and it was found that race was not an independent prognostic factor in OCS [28]. Patients’ characteristics must be viewed holistically in appreciation of socioeconomic and comorbid status, which may be more pronounced among different demographics. Nevertheless, ovarian cancer in England was not found to be associated with deprivation, although it is important to consider the variations in healthcare systems internationally, as the National Health Service (NHS) “free at the point of use” ethos is not universally applicable and financial cost could contribute to late presentation.

### Prognostic factors

One important factor associated with worse survival in patients with OCS is the advanced stage of the disease, according to the FIGO classification [13•, 29, 30]. A study reviewed 37 cases of OCS, with 70% of patients staged at III or IV, and reported a significant decrease in survival rates, dropping from 40 to 6% after a 5-year period [30]. It concluded that the early FIGO stage was an independent prognostic factor for OCS survival. A 2011 study discovered that patients with advanced OCS received poorer response rates to platinum-based chemotherapy compared to the cohort of patients with serous EOC [31], which is supported by other studies that noticed consistently worse survival in all stages of OCS compared with individuals diagnosed with high-grade serous ovarian cancer [10, 17]. Older age at diagnosis has also been identified as a poor prognostic factor in patients with OCS, alongside the presence of lymph node metastasis [29, 31, 32]. A 2010 study reported significantly improved survival in patients who underwent lymphadenectomy, reducing the mortality risk by 34% in such patients compared with those who did not have lymphadenectomy [29]. Additionally, the presence of residual disease following debulking surgery is linked to reduced survival rates and lower disease-free survival, mainly due to an increased risk of recurrence. Therefore, achieving optimal surgical cytoreduction is a crucial prognostic factor that significantly impacts survival outcomes [33–35].

Histopathologic features may represent an additional predictor of prognosis. Various studies have supported the hypothesis that heterologous features—elements that are not usually present in the ovary—are correlated with less favourable outcomes [34–37], whilst others have contradicted these findings [30, 33, 38, 39]. One study investigated the effects of epithelial and stromal tumour components on advanced OCS and concluded that tumours with more than 25% stromal components significantly resulted in worse outcomes [39]. Increased expression of vascular endothelial growth factor (VEGF), a potent angiogenic factor, and an increased number of small vessels are important in predicting poor survival [40]. High expression of the mutated form of tumour suppressor protein p53 has been frequently observed in gynaecological malignancies, particularly in OCS [41, 42]. Overexpression of p53 alongside Wilms tumour 1 (WT1) protein resulted in reduced OS [43]. Carter et al. discovered that the 20-year survival probability of individuals with no expression of either p53 or WT1 was 67.7%, which drastically reduced to 6.5% in those with both WT1 and p53 expression [43]. Whilst there is insufficient data on the influence of the Ki67 antigen expression on OCS survival, one study concluded that the 5-year survival in the highly-expressed Ki67 group was 15.9% compared to 36.4% in the low-expressed group but was deemed statistically insignificant [38]. There are various studies investigating the expression of Ki67 in other gynaecological malignancies, such as uterine carcinosarcomas [44–47]. Finally, cancer antigen 125 (CA 125) levels were found to be elevated in 74% of OCS patients but deemed to be statistically insignificant for survival prediction [14]. However, a study conducted by Nazari et al. reported a statistically significant relationship between CA 125 levels and OCS staging, although the authors acknowledged its limitations as a sole prognostic tool [48].

### Therapeutic developments

#### Chemotherapy

Paclitaxel and carboplatin (PC) have been widely used as combined chemotherapy in the management of EOC [49], but the most effective regimen for OCS is not yet known. For the treatment of OCS, a 2022 randomised controlled trial (RCT) investigated the efficacy of PC compared to paclitaxel and ifosfamide; longer OS (30 vs. 25 months) and progression-free survival (PFS) (15 vs. 10 months) were reported in the PC group [50]. However, this trial explored both uterine carcinosarcoma (UCS) and OCS, enrolling a large group of 449 eligible patients with UCS but only 90 eligible OCS patients. The results regarding OCS specifically were reported to be determined with limited precision and found to be statistically insignificant. A phase II RCT (MITO-26) recruited 45 patients, with advanced or recurrent OCS (*n* = 32) and UCS (*n* = 13), to assess the safety and efficacy of trabectedin across seven centres in Italy [51]. To be eligible for the study, patients needed to have a disease that was not controllable through surgery and radiotherapy. Additionally, they were required to have received a maximum of two lines of chemotherapy prior to the commencement of the study. Regarding adverse events, 8 patients experienced decreased white cell and neutrophil counts, 6 patients had increased alanine aminotransferase (ALT) and aspartate aminotransferase (AST), and 2 patients died directly as a result of drug-related haematological toxicity. The objective response rate (ORR) was 11.9%, with a median PFS and OS of 2.01 and 4.64 months, respectively. However, the MITO-26 study acknowledges that, due to the current absence of a standardised treatment, a comparator treatment cannot be utilised to assess the drug’s efficacy. Furthermore, the study did not measure the primary endpoint for OCS patients exclusively, and therefore, trabectedin as monotherapy in OCS management needs further exploration.

A retrospective cohort study explored patients who were experiencing persistent or recurrent gynaecological carcinosarcomas and investigated the impact of second-line chemotherapy on OS in both UCS and OCS [52]. Among 46 patients, only 7 were confirmed OCS patients who received second-line chemotherapy regimens of adriamycin (*n* = 1), carboplatin and docetaxel (*n* = 4), PC (*n* = 1), and other regimens (*n* = 1). The decision to receive monotherapy or combined chemotherapy was determined by whether the patient had a treatment interval of less or greater than 180 days. PFS and median OS were calculated as 6.9 and 11.9 months respectively, but the results were not statistically significant. The study had an inadequate sample size to confirm whether combination or monotherapy was more efficient than the other, and further work is needed to establish first- and second-line therapies for OCS.

#### Surgery and Chemotherapy

Optimal cytoreductive surgery followed by chemotherapy has been frequently considered in the primary management of the disease, despite no available RCTs [53]. A retrospective study reviewed a cohort of patients (*n* = 267) from 1999 to 2021 who were diagnosed with peritoneal dissemination from tubo-ovarian malignancies and had undergone cytoreductive surgery (CRS) and hyperthermic intraperitoneal chemotherapy (HIPEC) [54]. Sixteen out of the 267 patients had confirmed OCS with advanced disease (FIGO stage III or above) and received adjuvant chemotherapy with either cisplatin, a combination of cisplatin and doxorubicin, or melphalan. Management with CRS and HIPEC reported no 90-day mortality and was associated with low rates of malignant bowel obstruction; nevertheless, its significance on the median OS cannot be determined until larger cohort studies with longer follow-ups are performed. Interestingly, another retrospective study conducted by Nizam et al. identified 27 OCS patients and investigated the impact of adjuvant platinum-based chemotherapy on OS [53]. The study concluded that the OS in patients who underwent adjuvant chemotherapy was significantly improved compared to those who did not, and the stage of disease did not affect the outcome. Further support came from a cohort study conducted by Hollis et al. in 2022, which revealed an extended OS among OCS patients who underwent debulking surgery followed by platinum-based adjuvant chemotherapy [10]. However, the investigators concluded that residual disease absence after debulking surgery and early-stage disease were both strongly associated with improved survival [10].

A case series conducted by Sethi et al. studied patients receiving adjuvant chemotherapy with PC after a total abdominal hysterectomy (TAH) with bilateral salpingo-oophorectomy (BSO) and complete lymph node dissection (LND) [55]. The paper concluded that cytoreductive surgery with LND observed better survival compared to minimal surgery. Similarly, another patient underwent a TAH, bilateral adnexectomy, omentectomy, and lymphadenectomy with no residual disease, followed by PC treatment [56]. The published report noted that the patient was disease-free when followed up at 45 months. Additionally, a large French retrospective multi-centre study included 425 patients with gynaecological carcinosarcomas, of whom 112 women had advanced OCS (FIGO stage III or above) [57]. Analysis revealed that OCS patients who underwent both upfront primary debulking and interval cytoreductive surgery followed by front-line chemotherapy had significantly better PFS.

Kozłowski et al. explored 14 patients with primary OCS. Among them, 10 patients underwent surgery and adjuvant platinum-based chemotherapy [58]. The authors reported one particular patient who had a recurrence 29 months after diagnosis, after initially being treated with platinum and paclitaxel chemotherapy prior to surgery, subsequently received adjuvant chemotherapy with the same regimens, and had an OS of 46 months. However, the results of other patients were not discussed, and the impact of surgery and chemotherapy or surgery alone on the OS could not be determined. Interestingly, Heinzelmann-Schwarz et al. conducted a retrospective study looking at the efficacy of adjuvant chemotherapy in various subtypes of endometrial and ovarian cancer, including 17 OCS patients [59]. The study found that OCS had a higher chemotherapy sensitivity (73.9%) to a combination of carboplatin and anthracyclines than carboplatin/taxane-based (39.5%) and carboplatin/alkylating combinations (24.2%), although statistical significance was not discussed. Further studies to confirm the value of carboplatin/anthracyclines as the optimal adjuvant chemotherapy must be performed. A case report mentioned one particular patient with advanced OCS who underwent neoadjuvant chemotherapy (NACT), followed by non-curative unilateral resection of the appendicular organ, total omentectomy, and adjuvant chemotherapy [60]. She experienced recurrence roughly 21 months after this initial surgery and received aggressive tumour resection of the para-aortic and/or pelvic tumours, and replacement of the abdominal aorta and/or iliac arteries with a synthetic arterial graft. Unfortunately, there were confirmed lung and mediastinal lymph node metastases 1.3 years later, and the patient died 5.9 years after diagnosis. It is interesting to note that this patient had a greater OS compared to other studies reviewed, suggesting the benefits of radical surgical intervention in OCS.

Zuhdy et al. reviewed three cases of OCS associated with axillary lymph node metastasis who either received NACT or adjuvant therapy, but the median OS and efficacy of individual treatment approaches could not be determined due to patients being lost to follow-up [61]. To decide on the best suitable treatment for OCS with axillary lymph node metastasis, multi-centre studies with large sample sizes are required.

#### Surgery, Chemotherapy, and Targeted Therapy

A recently published report described a patient diagnosed with rapidly progressive OCS who had undergone uncomplicated embryo transfer 16 days prior [62]. The patient received 4 cycles of NACT with PC every 3 weeks. Interval computed tomography (CT) imaging demonstrated reduced peritoneal disease and resolved ascites, and subsequently, the patient underwent optimal debulking surgery. The patient received 2 more cycles of adjuvant platinum-based chemotherapy with the addition of bevacizumab, which was continued as maintenance therapy. At the time of writing, the most recent CT scan showed no evidence of disease, but this report does raise the question of the long-term effects of assisted reproduction technology on developing OCS. Nevertheless, NACT may have an important role in optimising debulking surgery in OCS, thus improving patient outcomes, which has also been supported by Liu et al. [63]. A case report discussed a 61-year-old female with advanced OCS who underwent extensive cytoreductive surgery including a hysterectomy, bilateral adnexectomy, partial resection of the rectum and sigmoid colon, and LND [21]. She subsequently received 6 cycles of chemotherapy with PC, plus bevacizumab during cycles 3 to 6. HRD testing was positive, so it was decided to put the patient on bevacizumab and the PARP inhibitor niraparib, but the patient developed severe myelosuppression, and thus, she received oral niraparib as maintenance therapy instead. Six months after the sixth cycle of chemotherapy, the CA 125 levels dropped from 263.4 to 4.55 U/ml, reducing to normal levels. Long-term survival data were not reported. However, during the six-month follow-up, imaging assessments revealed no signs of tumour recurrence or disease progression.

Ovarian teratoid carcinosarcoma (OTC) is an even rarer type of OCS, with the addition of an immature neuroectodermal component [64, 65]. Two case reports investigated two women with confirmed OTC [64, 65]. A 60-year-old female was diagnosed with stage IC3 OTC and underwent radical TAH-BSO, pelvic and para-aortic lymphadenectomy, and subtotal omentectomy [64]. Two months post-surgery, the patient experienced recurrent disease with multiple liver and bone metastases on positron emission tomography CT scan (PET-CT). However, following 4 cycles of vincristine, actinomycin, and cyclophosphamide chemotherapy, a complete response was achieved. Subsequently, 2 months after completing chemotherapy, the patient experienced recurrent peritoneal dissemination. The treatment involved 6 cycles of PC combined with bevacizumab, resulting in a partial response [64]. Unfortunately, 2 weeks after completing chemotherapy, the patient died due to complications from bevacizumab. Similarly, Fox et al. reported a 55-year-old female who was diagnosed with stage IC OTC and underwent optimal cytoreductive surgery with TAH-BSO, pelvic lymphadenectomy, and omentectomy [65]. The patient recurred 1 month after the surgery and was then given 6 cycles of ifosfamide and paclitaxel, followed by 3 cycles of carboplatin, gemcitabine, and bevacizumab. Chemotherapy was discontinued after a follow-up CT scan confirmed disease progression; therefore, the patient received 2 cycles of nivolumab, which was not effective. Due to disease progression, the patient was given a 5-day cycle of bleomycin, etoposide, and cisplatin. However, 1 week later, the patient died due to neutropenic sepsis, prior to receiving cycle two. The OS was 14 months since the initial surgery. Both patients experienced recurrent disease 1 to 2 months after surgery, confirming the aggressive nature of the disease. Furthermore, a *PIK3CA* mutation was detected in both patients, suggesting that targeting the PIK3CA pathway may be valuable in the treatment of OTC.

### Future directions

Table 1 summarizes ongoing investigations for the management of OCS. The emphasis of current trials is mostly centred on establishing the role of targeted therapies [13•]. Indeed, current clinical trials encompass a range of treatment approaches, either as singular interventions or in combination. These strategies involve PD-1 [66–72], PD-L1 [73], VEGF [71, 73, 74], PARP [72, 73], cytotoxic T-lymphocyte-associated antigen 4 (CTLA-4) [69], mTOR [75], or complement component 3 (C3) [74] inhibitors.

**Table 1:** Ongoing studies investigating the treatment options for OCS

Clinical trial	Condition/disease	Study type	Intervention/treatment	Estimated enrolment	Primary outcome	Time frame to measure primary outcome
NCT05619913 [66]	OCS and UCS	Phase II, randomised, interventional	Arm 1: eribulin Arm 2: eribulin + pembro	30	CBR	12 weeks
NCT03740165 [67]	Ovarian cancer, fallopian tube cancer, and peritoneal neoplasms	Phase III, randomised, interventional	Arm 1: pembro + ola Arm 2: pembro + placebo for ola Arm 3: placebo for pembro + placebo for ola	1367	PFS	Up to 57 months
NCT05265793 [68]	PD-1 immunotherapy, VEGFR-TKI, sarcomatoid carcinoma, and carcinosarcoma	Phase II, interventional	Camrelizumab + apatinib	45	ORR	An average of 1 year
NCT04969887 [69]	Advanced biliary tract cancer, neuroendocrine tumours, female reproductive system neoplasm, and MSI-H solid malignant tumour	Phase II, interventional	Ipilimumab + nivolumab	240	CBR PFS	12 weeks 6 months
NCT05224999 [70]	Recurrent/metastatic carcinosarcoma	Phase II, interventional	Nivolumab	28	PFR	6 months
NCT05044871 [71]	Ovarian cancer Arm 4 and 5: mucinous ovarian carcinoma, OCS	Phase II, non-randomised, interventional, five-arm study	Arm 4: tislelizumab + bev + nab-paclitaxel Arm 5: bev + nab-paclitaxel	160	ORR	Up to 3 years
NCT03651206 [72]	OCS and ECS	Phase II/III, randomised, interventional	Arm 1: niraparib Arm 2: niraparib + dostarlimab Arm 3: standard CTH	196	RR OS	4 months 1 year
NCT03737643 [73]	Advanced ovarian cancer	Phase III, randomised, interventional	Arm 1: bev, placebo ola, durva placebo, PC Arm 2: bev, placebo ola, durva, PC Arm 3: bev, durva, ola, PC Arm 4: tBRCAm cohort: bev, ola, durva, PC	1404	PFS	~ 4 years
NCT04919629 [74]	FT carcinosarcoma, FT AdenoCa, FT serous AdenoCa, OCS, ovarian clear cell AdenoCa, ovarian endometrioid AdenoCa, ovarian serous AdenoCa, primary peritoneal AdenoCa, recurrent FT carcinoma, recurrent ovarian carcinoma, and recurrent primary peritoneal carcinoma	Phase II, randomised, interventional	Arm 1: pegcetacoplan, pembro Arm 2: pegcetacoplan, pembro and bev Arm 3: bev	40	Accumulation of effusion	Up to 3 years
NCT03648489 [75]	Ovarian cancer, ovarian neoplasms, OCS, ovarian serous adeno, ovarian endometrioid adeno, ovarian clear cell adeno, FT cancer, FT neoplasms, primary peritoneal carcinoma, and primary peritoneal serous adeno	Phase II, randomised, interventional	Arm 1: paclitaxel Arm 2: paclitaxel and sapanisertib (TAK228)	134	PFS	12 months

*OCS*, ovarian carcinosarcoma; *UCS*, uterine carcinosarcoma; *pembro*, pembrolizumab; *CBR*, clinical benefit rate; *ola*, olaparib; *PFS*, progression-free survival; *PD-1*, programmed cell death; *VEGFR*, vascular endothelial growth factor receptor; *TKI*, tyrosine kinase inhibitor; *ORR*, objective response rate; *MSI-H*, microsatellite instability-high; *bev*, bevacizumab; *PFR*, progression-free rate; *ECS*, endometrial carcinosarcoma; *CTH*, chemotherapy; *RR*, response rate; *OS*, overall survival; *durva*, durvalumab; *PC*, paclitaxel + carboplatin; *tBRCAm*, tumour BRCA mutation; *FT*, fallopian tube; *AdenoCa*, adenocarcinoma

The majority of ongoing clinical trials involve PD-1/PD-L1 inhibitors. PD-1/PD-L1 is a receptor-ligand system and can prevent anti-tumour immune responses [76–78]. T cells of the immune system greatly express PD-1, whereas cancer cells and antigen-presenting cells express PD-L1 [78, 79]. Additionally, PD-L1 is highly expressed in ovarian cancer, and it is associated with tumour-infiltrating lymphocytes, cancer stem cell populations expressing CD44 (a cell-surface glycoprotein contributing to metastasis), and other stem cell markers [80]. Inhibiting PD-L1 via immune checkpoint inhibitors, such as durvalumab, may downregulate stem cell populations associated with cancer recurrence [80]. The efficacy of PD-1/PD-L1 inhibitors underwent a systematic review and meta-analysis by Chen et al., based on 91 phase I–III clinical trials of various cancers, including those of gynaecological origin [81]. This meta-analysis demonstrated that PD-1/PD-L1 inhibitors in combination with chemotherapy had a statistically significantly higher ORR when compared to immunotherapy alone.

As promising as this emerging therapy may appear, data regarding the effectiveness of immunotherapy in OCS are limited. The JAVELIN Ovarian 100 study of 998 patients—including an unspecified number of OCS patients—evaluated the PD-L1 inhibitor avelumab as a combination therapy versus maintenance therapy after PC chemotherapy [82]. The trial was halted prematurely as the interim analysis revealed that the addition of the checkpoint inhibitor avelumab to chemotherapy did not result in an improvement in PFS. The JAVELIN Ovarian 200 study enrolled a total of 566 patients—including 6 patients with OCS—who had platinum-resistant disease and had undergone no more than 3 previous therapies [83]. These patients were randomised into different groups receiving avelumab, pegylated liposomal doxorubicin (PLD), or a combination of treatments. The limited number of OCS patients in the sample size prevented the evaluation of response measurements for this specific population. Regarding ovarian cancer as a whole, the results indicated that neither avelumab alone nor avelumab in combination with PLD showed improvements in PFS or OS compared to PLD alone. This may suggest that the combination of chemotherapy and immunotherapy might not be the most effective treatment approach for OCS patients. Patient selection could be improved through the analysis of tumour histology, HRD status, and previous treatments [84, 85]. There is currently an inadequacy of large-scale studies evaluating HRD rates among OCS patients. A recent study demonstrated that HRD testing results can be obtained in a timely manner for making therapeutic decisions by ensuring maximal tumour tissue is collected during the initial diagnosis, thereby ensuring optimal tumour quality and quantity for testing [84]. To enhance accuracy, it was observed that conducting additional testing for *BRCA* mutations reduces the occurrence of HRD testing failures and should be routinely performed.

Immunotherapy has played a pivotal role in enhancing the outcome of several malignancies, including non-small cell lung cancer, melanoma, bladder cancer, and kidney cancer [86–90]. Nevertheless, the application of targeted therapies for OCS is primarily supported by preclinical data, as findings from clinical trials have been disappointing [1]. The ultimate objective is to achieve personalized immunotherapy, enabling tailored treatments for different OCS patients with distinct characteristics, thus avoiding needless side effects and minimizing healthcare expenses [1].

## Conclusion

OCS is a rare and aggressive malignancy, and its aetiology remains poorly understood. The rarity of the disease poses challenges in identifying the most effective therapeutic strategies to enhance patient outcomes. Given its unique histology and poorer survival rates compared to EOC, OCS may require a distinct approach. However, the lack of sufficient studies makes it difficult to establish a standardised treatment plan for OCS patients, as most existing research primarily consists of case studies involving various treatment regimens without appropriate comparator treatment groups. The best available evidence continues to support cytoreductive surgery, followed by platinum-based chemotherapy. Despite the promising potential of the PD-1/PD-L1 receptor-ligand system as an immuno-oncology target, there is conflicting evidence concerning the effectiveness of immune checkpoint inhibitors in OCS. This disparity highlights the necessity for more rigorous studies to gain a comprehensive understanding of their role in treating the OCS. HRD testing may identify specific patient populations for targeted therapies. Given the heterologous nature of OCS, maximal tumour tissue should be obtained during the initial intervention to achieve optimal tumour quality and quantity for HRD testing.

## Data Availability

Not applicable.
